# Predators or Herbivores: Cockroaches of Manipulatoridae Revisited with a New Genus from Cretaceous Myanmar Amber (Dictyoptera: Blattaria: Corydioidea)

**DOI:** 10.3390/insects13080732

**Published:** 2022-08-15

**Authors:** Xinran Li, Diying Huang

**Affiliations:** State Key Laboratory of Palaeobiology and Stratigraphy, Center for Excellence in Life and Paleoenvironment, Nanjing Institute of Geology and Palaeontology, Chinese Academy of Sciences, Nanjing 210008, China

**Keywords:** Blattodea, Corydiidae, ecology, flower-visiting, fossil, kleptoparasite, systematics

## Abstract

**Simple Summary:**

There are many more wild cockroaches than household species, but they mostly live a similar life to the latter, feeding on dead and inactive organic material, and hiding in confined spaces. Most of the fossil cockroaches are similar to modern ones in their general appearance, so the lifestyle of the fossil species is probably similar to that of modern cockroaches. Years ago, a unique fossil cockroach, *Manipulator modificaputis* Vršanský and Bechly, 2015, was discovered from Cretaceous Myanmar amber. This species somewhat resembles mantises and was speculated to be a predator. However, this hypothesis is debatable. In the present study, we redescribe *Manipulator modificaputis* based on new material, and describe a closely related new species, *Manipulatoides obscura* gen. & sp. nov. We discuss the feeding habit of these species, and consider that they tend to live around and feed on flowers (as occasionally do some modern cockroaches), instead of hunting for prey. So far, no cockroaches specialized in predation have been affirmed.

**Abstract:**

*Manipulator modificaputis* Vršanský and Bechly, 2015 (Manipulatoridae, Corydioidea) is a purported predatory cockroach from Cretaceous Myanmar amber, based on a single male. It is distinctive by the nimble head, elongate pronotum and legs, and particularly by the extraordinarily long maxillary palpi. In the present study, we redescribe *Manipulator modificaputis* based on six new fossils including males and females, and comment on the original description. The closely related *Manipulatoides obscura* gen. & sp. nov. is proposed on the basis of five fossils, including males and females. It differs from *Manipulator* in weaker spination of the legs, including the type-C forefemoral spination instead of the type-A of *Manipulator*. Some undetermined adults and nymphs are also described. We discuss the ethology of Manipulatoridae and speculate that they might feed on flowers. They are unlikely to be specialized predators since they lack necessary weaponry for capturing prey; in contrast, their unique morphotype appears to be suitable for efficient foraging and locomotion amid flowering twigs. The possibility of being kleptoparasites of the spider-web is also discussed. In addition, regenerated four-segmented tarsi are found from the new species.

## 1. Introduction

The Cretaceous amber from northern Myanmar preserves an amazing diversity of insects, which include various cockroaches [1,2,3]. Among them is a unique cockroach, *Manipulator modificaputis* Vršanský and Bechly, 2015, which has a saddle-like pronotum and significantly elongate appendages, particularly the maxillary palpi [4]. The family Manipulatoridae was proposed at the same time. Vršanský and Bechly considered *M. modificaputis* to be predatory based on the distinctive morphotype [4]. However, this conjecture is debatable. In the present study, 21 new fossils allow a re-investigation of Manipulatoridae. As a result, we redescribe *Manipulator* and propose a new genus *Manipulatoides*. The original description of *Manipulator* and the ethology of the family are discussed.

## 2. Materials and Methods

The ambers are from deposits in the Hukawng Valley of northern Myanmar (see [5,6]). The age is considered to be around the Albian-Cenomanian boundary [6,7,8,9,10,11], likely latest Albian. Specimens are deposited at Nanjing Institute of Geology and Palaeontology (NIGP), Chinese Academy of Sciences, with accession numbers NIGP174194–174213 and NIGP174363.

To get a clearer view, the ambers were sanded with abrasive papers and polished with polishing powder. Photos were taken with the following devices: a Zeiss AxioZoom V16 stereoscope (Carl Zeiss Microscopy GmbH, Jena, Germany), a Zeiss SteREO Discovery V20 stereoscope (Carl Zeiss Microscopy GmbH, Jena, Germany), a Canon 5D II camera mounted with a Canon EF 100 mm f/2.8L IS USM Macro lens (Canon Inc., Tokyo, Japan), and a Zeiss AXIO Imager Z2 microscope (green fluorescence) (Carl Zeiss Microscopy GmbH, Jena, Germany). Photos of shallow depth of field were stacked using CombineZP 7.0 (Alan Hadley, Sheffield, UK; available from http://www.micropics.org.uk/ accessed on 1 June 2022) and Photoshop CC 2015 (Adobe Systems Incorporated, Delaware/San Jose, CA, USA); and optimized using Photoshop CC 2015. Micro-CT scanning was done with a Zeiss Xradia 520 Versa under a resolution of 3.5628–3.7128 μm (Carl Zeiss Microscopy GmbH, Jena, Germany). A total of four regions of three specimens were scanned, but unfortunately only the head of one specimen yielded satisfactory results. Three-dimensional reconstruction was performed in InVesalius 3.1.1 (Center for Information Technology Renato Archer, Campinas, Brazil; available from https://www1.cti.gov.br/pt-br/invesalius accessed on 1 June 2022) [12,13].

Morphological terminology largely follows Roth [14]; terms for wing venation follow Li et al. [15]. The unit of measurements is millimetre. The question mark in measurements denotes difficulties in measuring. Names of higher taxa are character-based, as clarified or defined in Li [16]. This publication and nomenclatural acts herein were registered in ZooBank, and the LSID for this paper is urn:lsid:zoobank.org:pub:C3289D53-B5A1-4F97-A869-EEAC62231BDE.

Abbreviations of wing veins. Posterior sector of Subcosta (ScP), Radius (R), anterior sector of Radius (RA), posterior sector of Radius (RP), Media (M), Cubitus (Cu), anterior sector of Cubitus (CuA), posterior sector of Cubitus (CuP), Postcubitus (Pcu), Vannal veins (V), anteriormost vannal vein along the vannal fold (V[1]), vannal veins that fill the vannus (V[s]).

## 3. Results

Systematic Palaeontology.

Order Dictyoptera Leach, 1815 *sensu* Ax, 1999.

Suborder Blattaria McKittrick, 1964 *sensu* Klass and Meier, 2006.

Superfamily Corydioidea de Saussure, 1864.

Family Manipulatoridae Vršanský and Bechly, 2015.

Re-diagnosis. Medium- to small-sized cockroach, with elongate pronotum and appendages. Head nimble, hypognathous in repose, wider than anterior half of pronotum and narrower than the posterior half of pronotum. Eyes bulging, reniform or subglobose, moderate size among cockroaches. Along the higher margin of antennal socket is a crescent fenestra (transparent area), above which is a brim-shaped protrusion. It is uncertain whether the fenestra and the ocellus are identical. The fenestra may be the ocellus itself or contains the ocellus as a part. Maxillary palpi longer than double head length. Pronotum longer than width, widest at posterior one-fifth. In macropterous species, tegmen length-width ratio approximately 10:3; vannus of hindwing small, folding over flatly, not fanwise. Basally between RP and M of hindwing is a short vein, seemingly a vestigial M branch rather than a cross-vein. Legs long, foreleg longer than body, hindleg almost double the length of foreleg. Males with hook-like phallomere on the left side. Female seventh sternum (subgenital plate) bivalvate, valvulae concealed.

Taxonomic placement and phylogenetic position. Vršanský and Bechly proposed Manipulatoridae based on *Manipulator* [4]. Although the higher classification of cockroaches is based largely on genital structures and oviposition behaviour [14,17], we agree that *Manipulator* represents a distinct family in view of its unique character set regardless of genital traits. Vršanský and Bechly listed many autapomorphies of Manipulatoridae [4]; however, we consider only three of them to be candidates for autapomorphies in Dictyoptera: saddle-like pronotum, extremely elongate maxillary palpi, and crescent fenestra (which encompasses ocellus or is ocellus itself) with a ‘brim’ above (=“ocelli with roof-like covering sheaths” therein). In addition, we suggest two more candidates for autapomorphy: one is the hypognathous head, and the other is the oblique vein basally between RP and M in the hindwing (see below for details).

Manipulatoridae is included in Blattaria due to the concealed female valvulae. Within Blattaria, Manipulatoridae resembles Corydioidea in the small and flat vannus of hindwing. The bivalvate female subgenital plate is also characteristic for some Corydioidea. Therefore, we include Manipulatoridae in Corydioidea. Manipulatoridae have some superficial similarities with Mantodea, but differ from the latter in many key characters, e.g., central ocellus, forelegs, and ovipositors.

**Figure 1:**
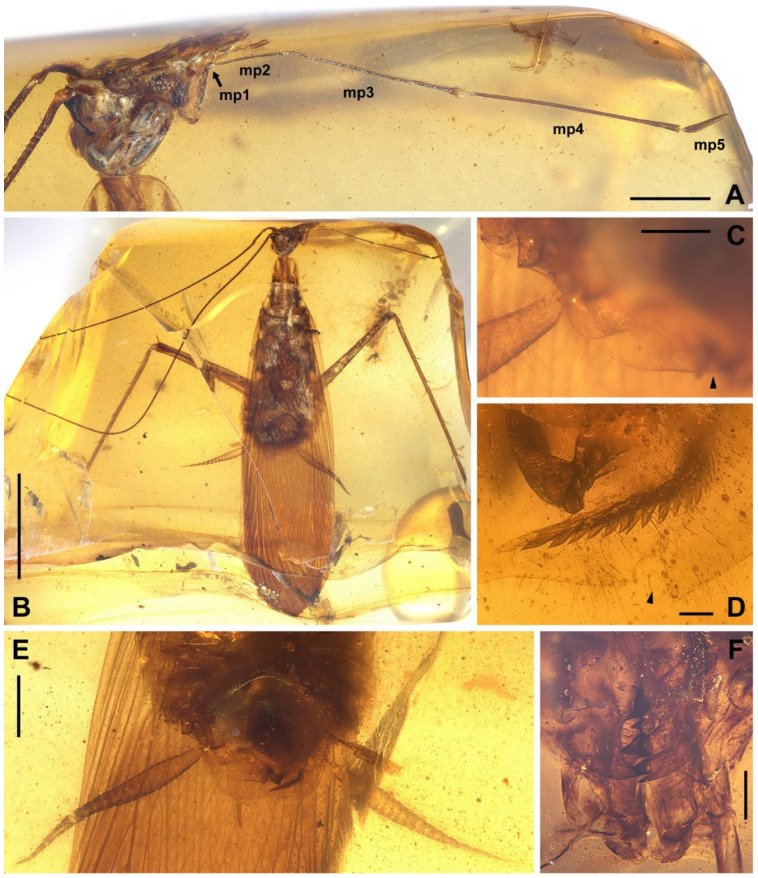
*Manipulator modificaputis* Vršanský and Bechly, 2015, NIGP174194, male. (**A**) Head, lateral view, showing the stretched maxillary palpus. (**B**) Dorsal view of the specimen. (**C**) Supra-anal plate, ventral view, with an arrowhead indicating the midpoint of the hind margin. (**D**) Partial phallus and subgenital plate, ventral view, with an arrowhead indicating the insertion of the lost left stylus. (**E**) Terminalia in ventral view; note that the left stylus is lost. (**F**) Partial mouthparts, showing mandibles; note that the labrum is impaired. Abbreviations: mp1–5, maxillary palpomere I–V. Scale bars: (**A**,**E**) = 1 mm, (**B**) = 5 mm, (**C**) = 500 µm, (**D**) = 100 µm, (**F**) = 200 µm.

Manipulatoridae share some traits with the extinct Mesozoic family Raphidiomimidae. Their habitus is elongate and agile, with the pronotum longer than width [18,19]. The maxillary palpi of Raphidiomimidae are elongate compared with extant cockroaches, although not that long as Manipulatoridae. Remarkably, the prognathous head of Raphidiomimidae and the hypognathous head of Manipulatoridae are not found from extant cockroaches, of which the head is opisthognathous in repose. Superficially, Manipulatoridae is intermediate between “traditional” cockroaches and Raphidiomimidae. However, Raphidiomimidae differ from Manipulatoridae in critical characters: large fanwise vannus of hindwing, and slightly exposed ovipositor. Interestingly, *Fortiblatta cuspicolor* Liang et al., 2009, a species placed in Raphidiomimidae, possesses the oblique vein basally between RP and M in the hindwing ([20]: Figures 1a and 3c–d).

### 3.1. Genus Manipulator Vršanský and Bechly, 2015

Type species. *Manipulator modificaputis* Vršanský and Bechly, 2015. Original monotypy.

Diagnosis. The forefemoral spination of *Manipulator* is type A, instead of type C of *Manipulatoides* gen. nov. The spination of mid- and hindfemora is also stronger than that of *Manipulatoides* gen. nov. In addition, two minor differences between the generic types might be of intergeneric taxonomic value, but currently considered as interspecific: (1), the ‘brim’ above the likely ocellar fenestrae is more protrudent in *Manipulator modificaputis* than in *Manipulatoides obscura* gen. & sp. nov., and (2), the vein basally between RP and M in the hindwing is stronger.

*Manipulator modificaputis* Vršanský and Bechly, 2015.

**Figure 2:**
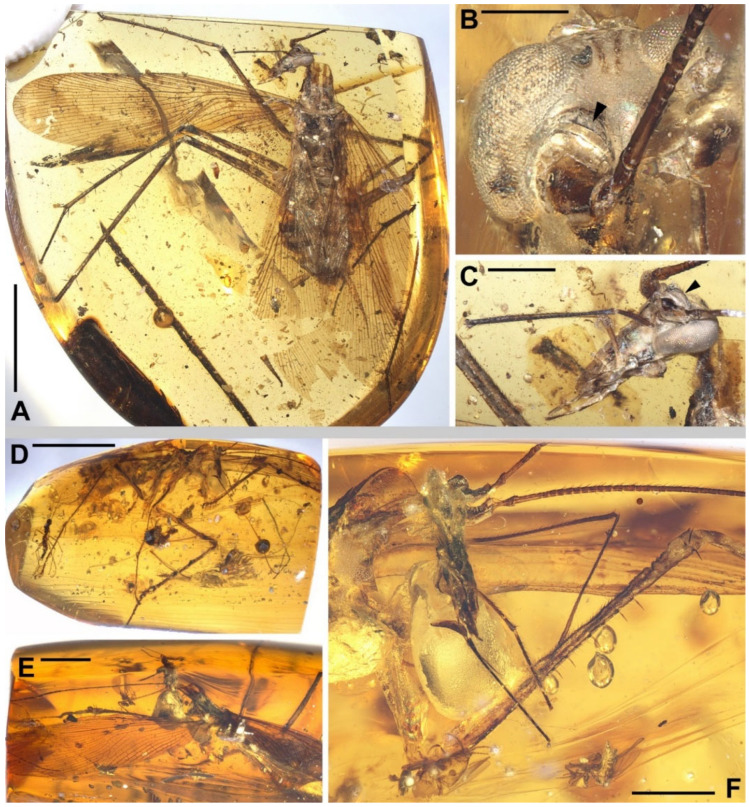
*Manipulator modificaputis* Vršanský and Bechly, 2015. (**A**–**C**) NIGP174195, female. (**A**) Dorsal view of the specimen. (**B**) Head, ventrocephalic view, with an arrowhead indicating the ‘brim’ above the fenestra, which is likely the ocellus. (**C**) Head, lateral view, with an arrowhead indicating the ‘brim’; note that the maxillary palpomeres III and IV are overlayed. (**D**–**F**) NIGP174196, male. (**D**) Lateral view of the specimen. (**E**) Dorsal view of the specimen, showing tegmina. (**F**) Lateral view of the anterior portion of the specimen, showing forefemoral spination, of which the spines are somewhat longer than that of other specimens (possibly because of preservation). Scale bars: (**A**,**D**) = 5 mm, (**B**) = 500 µm, (**C**,**F**) = 1 mm, (**E**) = 2 mm.

**Figure 3:**
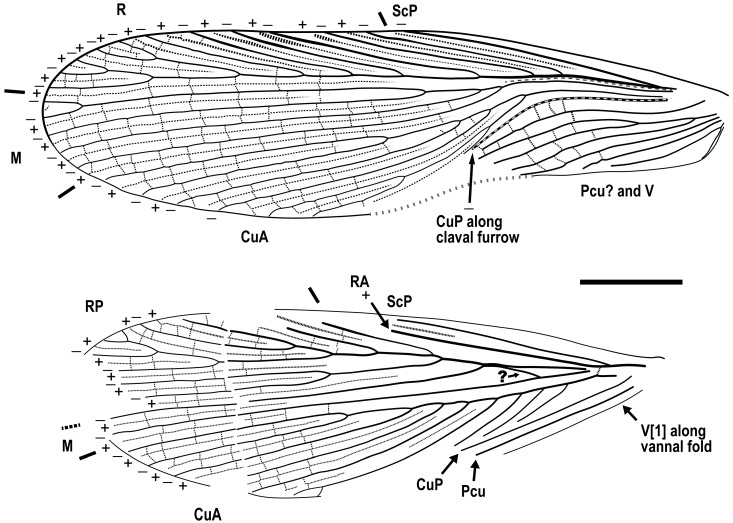
Wings of *Manipulator modificaputis* Vršanský and Bechly, 2015, NIGP174195 in dorsal view, left forewing and left hindwing. Densely dotted lines are intercalary veins and cross-veins; positive (+) and negative signs (–) indicate convex and concave veins, respectively. Dashed lines in the forewing are furrows. Grey barred line depicts the outline distorted by legs (see Figure 2A). Abbreviations of veins are explained in Materials and Methods. Scale bar = 2 mm.

**Figure 4:**
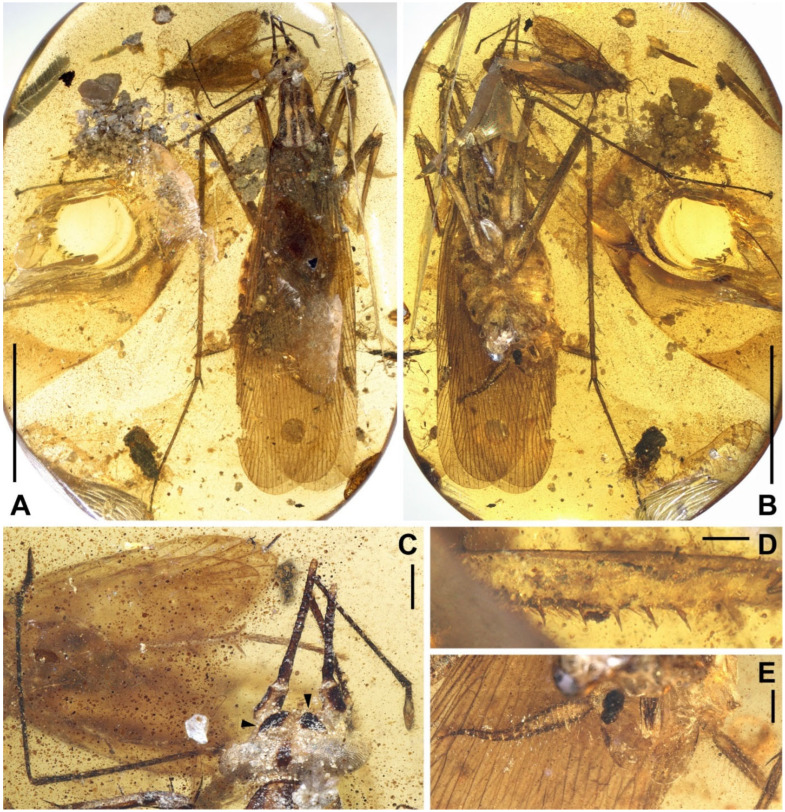
*Manipulator modificaputis* Vršanský and Bechly, 2015, NIGP174197, female. (**A**) Dorsal view. (**B**) Ventral view. (**C**) Head, dorsal view, with arrowheads indicating the ‘brims’ above the fenestrae, which are not visible from this perspective. (**D**) Spination of right forefemur, with six spines observable. (**E**) Incomplete terminalia in ventral view, showing exposed valvulae due to the absence of subgenital plate. Scale bars: (**A**,**B**) = 5 mm, (**C**,**E**) = 500 µm, (**D**) = 200 µm.

**Figure 5:**
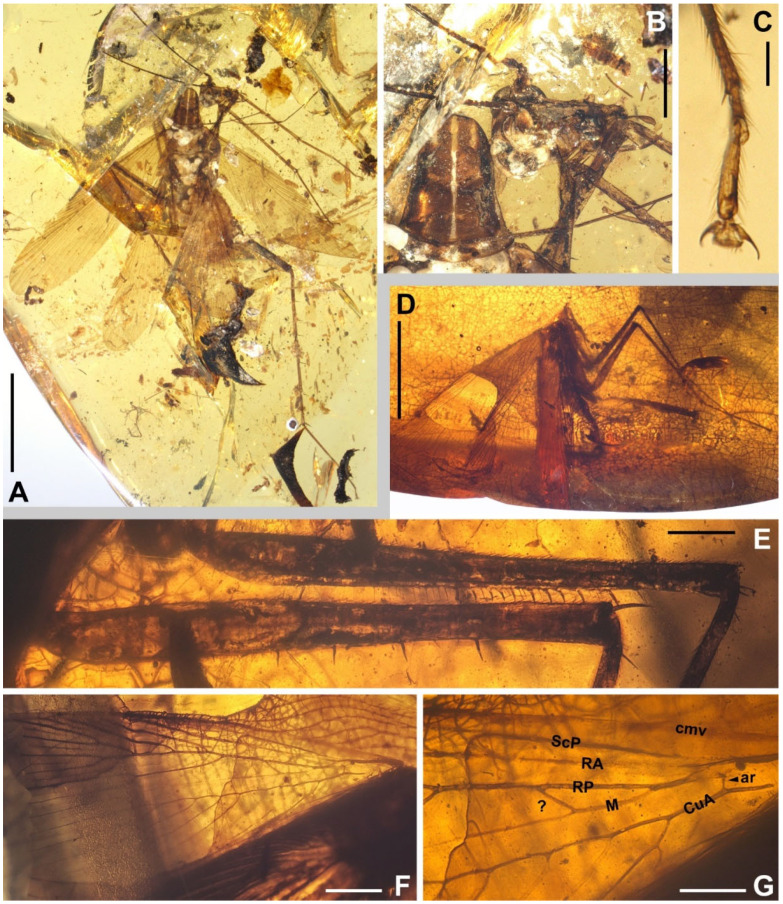
*Manipulator modificaputis* Vršanský and Bechly, 2015. (**A**–**C**) NIGP174199, sex unknown but probably male judging from its smaller size. (**A**) Dorsal view of the specimen. (**B**) Head and pronotum, dorsal view. (**C**) Hindtarsus and pretarsus. (**D**–**G**) NIGP174200, sex unknown. (**D**) lateral-dorsal view. (**E**) Right forefemur and midfemur. (**F**,**G**) Left hindwing with a close-up of the base, dorsal view; see the text for discussion about the question-marked vein. Abbreviations: ar, arculus; cmv, costo-marginal vein; other abbreviations of veins are explained in Materials and Methods. Scale bars: (**A**,**D**) = 5 mm, (**B**,**F**) = 1 mm, (**C**) = 200 µm, (**E**,**G**) = 500 µm.

Materials. Six non-types, NIGP174194–174197, NIGP174199–174200 (Figure 1, Figure 2, Figure 3, Figure 4 and Figure 5).

Redescription. Based on the materials in the present study. **Body** medium- to small-sized, head and thorax elongate, abdomen normal. Body length excluding head 9.1–9.3 (male), 10.4–10.9 (female). **Head** length 2.0–2.2. Vertex with a thick dark longitudinal stripe (Figure 4C) or a pair of thin stripes (Figure 2B). The ‘brims’ above fenestra (likely ocellus) very protrudent, dark-coloured (Figure 2B,C and Figure 4C). Antennae longer than body. Maxillary palpomere II–V lengths 0.75–0.83/1.9–2.1/2.1–2.6/0.45–0.60. **Pronotum** saddle-shaped, with variable dark markings but essentially four longitudinal stripes (remarkably in Figure 4A; cp. Figure 5B); supracoxal sulcus present; length 2.1–2.9, width of anterior half 1.2–1.4, of posterior half 1.8–2.1. **Wings** (Figure 3). Tegmen length 11.5–14.7 and width 2.8 (male), length 12.7–13.4 and width 3.7 (female). Costo-marginal vein (cmv) evenly running through the costal margin and ending around the apex of tegmen; ScP simple, almost reaching the midpoint of costal margin; R with pectinate branches proximally and nearly dichotomous branches distad; M and CuA similarly developed, branching pattern not specialized; R, M and CuA with 11/5/7? terminal branches; clavus longer than one-third of the wing length (probably reaching the midpoint of hind margin but uncertain due to difficulties in observation), claval veins essentially diagonal, towards the distal end of the clavus. Hindwing length 10.2–11.5 (female); ScP simple, almost reaching the midpoint of costal margin; RA simple, RP with ten or 11 terminal branches, radiating; basally between RP and M is a short oblique vein (question-marked in Figure 3 and Figure 5G), strong, seemingly an M branch rather than a cross-vein, therefore the identity of the longitudinal vein to the anterior is unclear; CuA with 6–7 complete branches that reach wing margin and 3–4 incomplete branches that do not; CuP and Pcu simple. Intercalary veins and cross-veins well-developed in tegmen and hindwing. **Leg** segment lengths (femur/tibia//tarsomere 1/2/3/4/5): male foreleg ?/5.0//1.9–2.0/0.78–0.88/0.34–0.47/0.11–0.12/0.30–0.33; female foreleg 5.9/5.6//?; female midleg 5.3–6.1/5.7–6.7//2.0/0.79/0.42/0.21/0.33; male hindleg 6.3–6.4/7.9–8.1//?; female hindleg 6.2–6.4/8.8–9.0//3.2/0.90/?. Coxae long, freely detached from body. Forefemoral spination type A1 according to Roth [14]. The anteroventral margin with a row of 6–11 short spines, interlaced with spinules or setae, preapically unarmed, and terminating in two spines arranged transversely, of which the dorsal one may be the genicular spine (Figure 2F, Figure 4D and Figure 5E). Forefemur with a terminal spine on the posteroventral margin, in addition to the aforementioned spines. Mid- and hindfemora with three terminal spines, one dorsal (genicular spine, largest), one anteroventral, and one posteroventral; additionally with 5–7 sparse large spines along the posteroventral margin. Apex of tibiae with five long spines. Additionally, foretibia with six spines sparsely arranged; midtibia with 11 (four anterodorsal, two anteroventral, two posterodorsal, and three posteroventral); and hindtibia with 19 (six anterodorsal, five anteroventral, five posterodorsal, and three posteroventral). Plantulae present at the apices of four proximal tarsomeres, the distal one distinctly larger whilst the others rather small. Claws symmetrical, not specialized, arolium moderate in size. **Male terminalia:** tergum X (supra-anal plate) nearly trapezoid, hind margin entire (Figure 1C); cercus length 3.5–3.6, with 24 or more segments, tapered, with dense long hairs. Sternum IX (subgenital plate) symmetrical, hind margin entire, slightly concave (Figure 1E); two styli far apart, interstylar distance ca. 0.88, right stylus length 0.43, left stylus missing (Figure 1D–E). Phallus with a serrated structure (Figure 1D). **Female terminalia:** cercus length 2.5–3.4, with 20 or more segments, tapered; sternum VII (subgenital plate) bivalvate apically, valvulae concealed.

### 3.2. Genus Manipulatoides Gen. Nov.

(LSID: urn:lsid:zoobank.org:act:4FB61ADB-D1AF-43C4-95BF-BE0E6CF7BC42)

Type species. *Manipulatoides obscura* gen. & sp. nov., original monotypy.

Etymology. Compounded from *Manipulator*, and -oides, likeness. Feminine.

Diagnosis. See the diagnosis of *Manipulator* above.

*Manipulatoides obscura* gen. & sp. nov.

(LSID: urn:lsid:zoobank.org:act:4BCACCB9-6A68-46B6-A642-BA1A44C44A7E)

Etymology. Latin adjective *obscura*, faintly seen, referring to the colour pattern of the new species.

**Figure 6:**
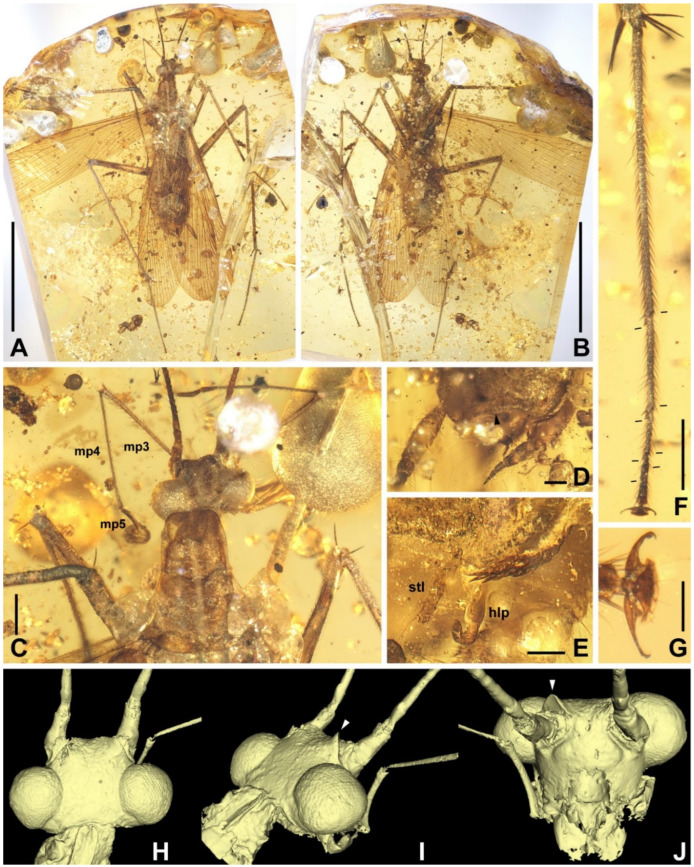
*Manipulatoides obscura* gen. & sp. nov., male holotype, NIGP174202. (**A**) Dorsal view. (**B**) Ventral view. (**C**) Head and pronotum, dorsal view. (**D**) Terminalia, ventral view, with an arrowhead indicating the hind margin of subgenital plate. (**E**) Details of the terminalia, showing hook-like phallomere and left stylus. (**F**) Hindtarsus, with bars marking the articulations between tarsomeres (right = dorsal, left = ventral). (**G**) Claws enlarged from F. (**H**–**J**) 3D reconstruction of the head from μ-CT, with arrowheads indicating the ‘brims’ above the likely ocellar fenestrae. (**H**) Dorsal view. (**I**) Lateral view, somewhat dorsocaudal. (**J**) Cephalic view. Abbreviations: hlp, hook-like phallomere; mp3–5, maxillary palpomere III–V; stl, stylus. Scale bars: (**A**,**B**) = 5 mm, (**C**,**F**) = 500 µm, (**D**) = 200 µm, (**E**,**G**) = 100 µm.

**Figure 7:**
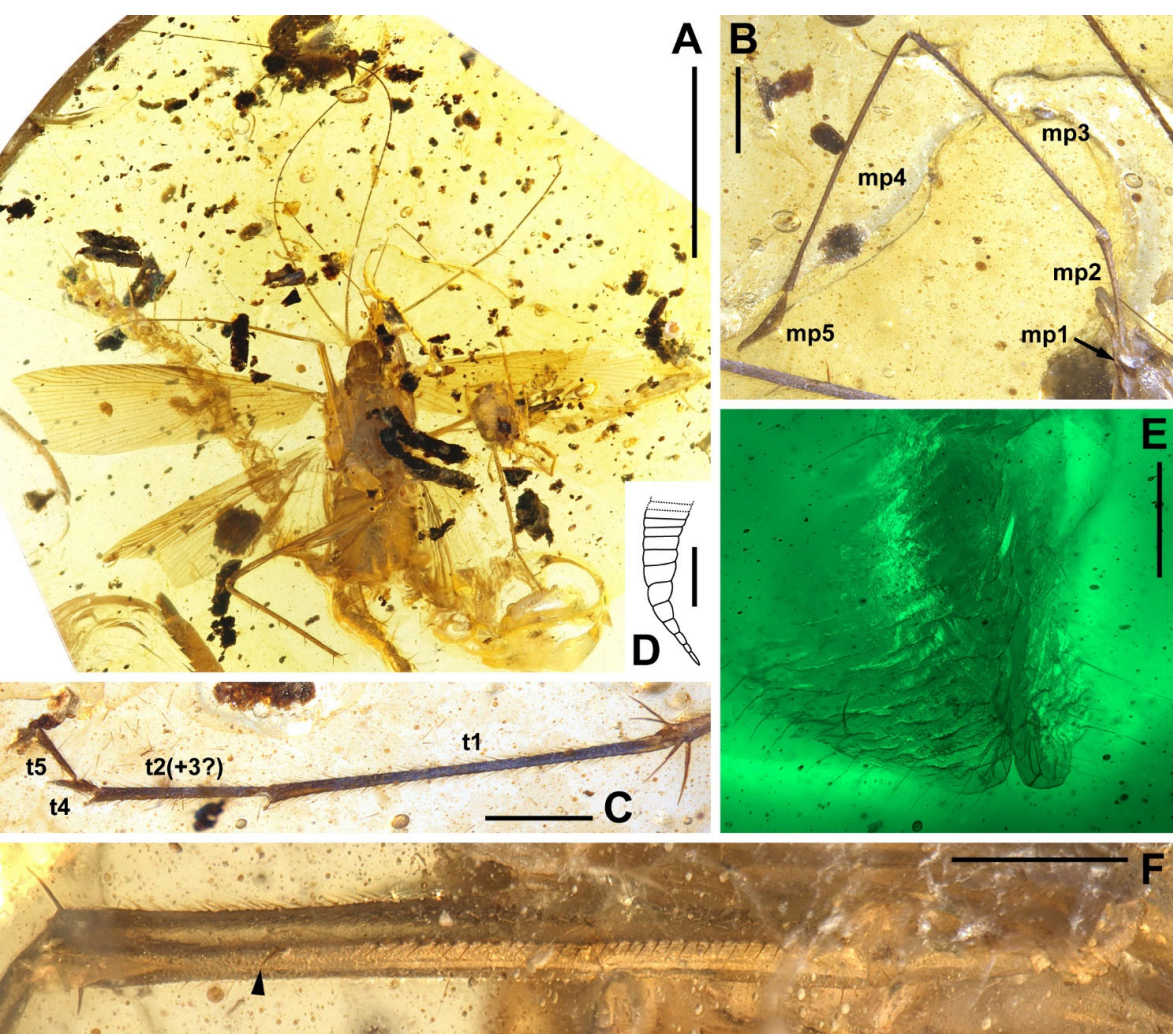
*Manipulatoides obscura* gen. & sp. nov., female allotype, NIGP174203. (**A**) Ventral view of the specimen. (**B**) Maxillary palpus. (**C**) Foretarsus. (**D**) Right cercus in ventral view. (**E**) Apex of seventh sternum (subgenital plate), note the valvation. (**F**) Right forefemur, with an arrowhead indicating a peculiar spine. Abbreviations: mp1–5, maxillary palpomeres I–V; t1–5, tarsomeres I–V. Scale bars: (**A**) = 5 mm, (**B**–**D**,**F**) = 500 µm, (**E**) = 200 µm.

**Figure 8:**
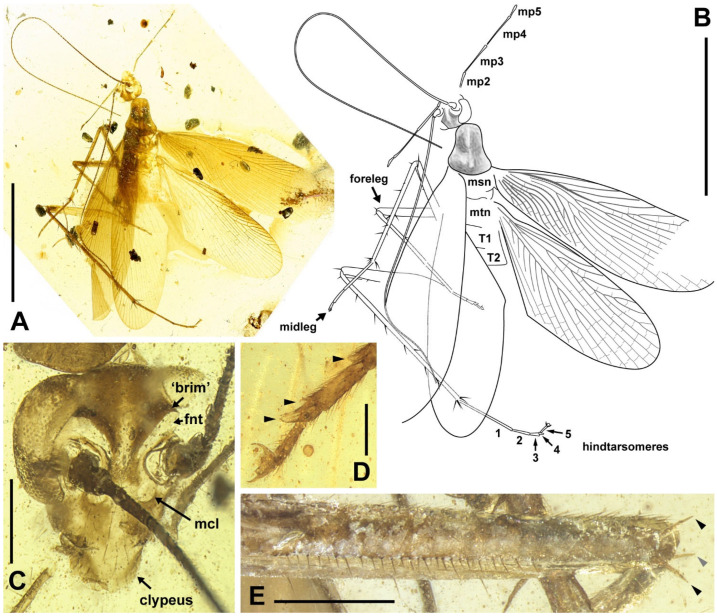
*Manipulatoides obscura* gen. & sp. nov., paratype, sex unknown, NIGP174204. (**A**,**B**) Dorsal view (see Figure 9 for the details of venation). (**C**) Head. (**D**) Apex of left foretarsus, with arrowheads indicating plantulae. (**E**) Left forefemur in anterior view; the two spines indicated by black arrowheads lie on the same surface as the spinules, and the grey arrowhead indicates the other side. Abbreviations: fnt, fenestra; mcl, macula; mp2–5, maxillary palpomeres II–V; msn, mesonotum; mtn, metanotum; T1 and T2, tergum I and II. Scale bars: (**A**,**B**) = 5 mm, (**C**,**E**) = 500 µm, (**D**) = 200 µm.

**Figure 9:**
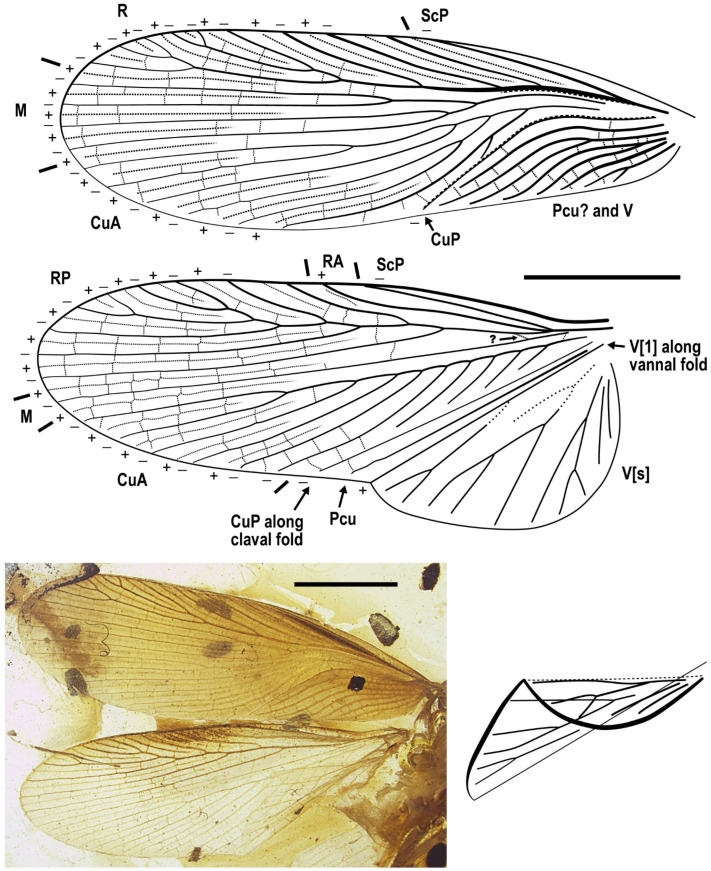
*Manipulatoides obscura* gen. & sp. nov., paratype, sex unknown, NIGP174204, right wings in ventral view. Densely dotted lines are intercalary veins and cross-veins. Positive (+) and negative signs (−) indicate convex and concave veins respectively, in relation to dorsal view. Dashed lines in the forewing are furrows. Sparse dotted lines in the hindwing are artificial, since this area is obscure in the specimen. Original preservation of the hindwing vannus is illustrated at bottom right, not to scale. Abbreviations of veins are explained in Materials and Methods. Scale bars = 2 mm.

**Figure 10:**
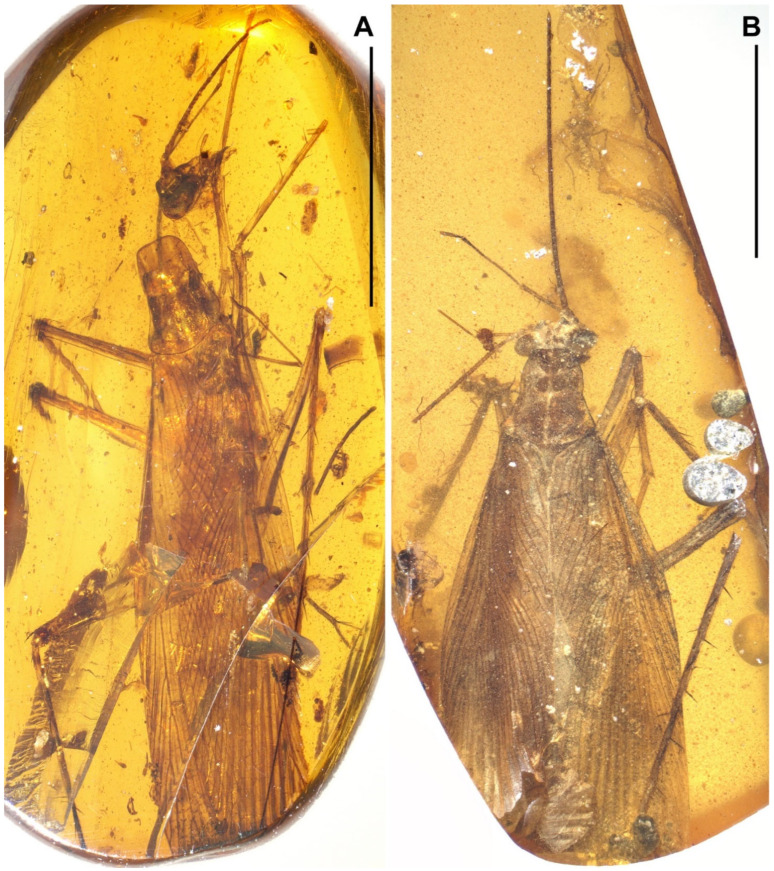
(**A**) *Manipulatoides obscura* gen. & sp. nov., paratype, sex unknown, NIGP174205, dorsal view. (**B**) *Manipulatoides obscura* gen. & sp. nov., non-type, sex unknown, NIGP174206, dorsal view.

Materials. Five adults. Holotype, male, NIGP174202 (Figure 6); allotype, female, NIGP174203 (Figure 7); paratype, sex unknown due the loss of abdomen, NIGP174204 (Figure 8 and Figure 9); paratype, sex unknown due the loss of abdomen, NIGP174205 (Figure 10A); non-type, sex unknown due to obscure terminalia, NIGP174206 (Figure 10B).

Type designation. The holotype NIGP174202 is a nearly complete specimen and the amber is clear enough to observe. The holotype of this family is a male, therefore, designating a male holotype for new species is desirable. Some characters of NIGP174202 are not shown in figures because of difficulties in photography (which hopefully can be overcame in the future), but strictly conform to the description and are comparable with the figures of other specimens. As the opposite sex, the allotype NIGP174203 supplements the holotype. The paratype NIGP174204 is readily observable, particularly the head and wings, but it cannot serve as the holotype or allotype because: (1) its terminalia are absent, and (2) the head and wings are also nearly completely preserved in the holotype and allotype, although there are temporary difficulties in thorough observation.

Description. **Head.** Vertex to frons with a pair of indistinct dark longitudinal stripes (Figure 6C), or the stripes with blurred margin filling the vertex and frons, to form a longitudinal light-coloured stripe between the fenestrae (Figure 8C); the ‘brims’ above the likely ocellar fenestrae dark-coloured, not as protrudent as in *Manipulator modificaputis* (Figure 6C,I,J and Figure 8C); eyes subglobose in holotype (Figure 6H–J) and reniform in paratype NIGP174204 (Figure 8C) but indiscernible in other specimens; antenna longer than body, lower margin of antennal socket with a light-coloured macula (Figure 8C). **Pronotum** saddle-shaped, with indistinct colour pattern, the midline somewhat lighter than background colour (Figure 6C, Figure 8A,B and Figure 10A,B); supracoxal sulcus present. **Wings** (Figure 9). Tegmen: cmv evenly running through the costal margin and ending around the apex of tegmen; ScP simple, reaching beyond one-third of the costal margin; R with pectinate branches proximally and nearly dichotomous branches distad; M and CuA similarly developed, branching pattern not specialized; R, M and CuA with 9/4/6 terminal branches; clavus longer than one-third of the wing length, claval veins almost ending at the hind margin of the wing. Hindwing: cmv tapering and ending around the apex of the wing; ScP simple, almost reaching the midpoint of costal margin; RA simple, RP nearly pinnate, with nine terminal branches; basally between RP and M is a short oblique vein (question-marked in Figure 9), weak, seemingly a vestigial M branch rather than a cross-vein; CuA with six complete branches that reach wing margin and four incomplete branches that do not; CuP and Pcu simple; V with 11 terminal veinlets. Intercalary veins and cross-veins well-developed in tegmen and hindwings. **Legs.** Coxae long, freely detached from body. Forefemoral spination type C1 according to Roth [14]; the anteroventral margin with a row of piliform spinules, terminating in two stout spines arranged transversely, of which the dorsal one may be the genicular spine (Figure 7F and Figure 8E). Note that a spine (possibly more) may occur in the spinule row (Figure 7F). Forefemur with a terminal spine on the posteroventral margin. Mid- and hindfemora with three terminal spines, one dorsal (genicular spine, largest), one anteroventral, and one posteroventral; without spines along the posteroventral margin or with 1–2 short slender spines in midfemur and/or 2–4 short slender spines in hindfemur. Apex of tibiae with five long spines. Additionally, foretibia with 3–4 sparsely arranged spines; midtibia with 8–9 (3 anterodorsal, 3 anteroventral, 1–2 posterodorsal, 1 posteroventral); hindtibia with 9–13 (4–5 anterodorsal, 1–2 anteroventral, 3–4 posterodorsal, 1–2 posteroventral). Plantulae present at the apices of four proximal tarsomeres, the distal one distinctly larger whilst the others rather small. Claws symmetrical, not specialized, arolium moderate in size. **Male terminalia:** cercus with 14 or more segments, tapered. Sternum IX (subgenital plate) symmetrical, hind margin entire, slightly concave, two similar styli far apart (Figure 6D). Hook-like phallomere and a serrated phallomere on the left (Figure 6E). **Female terminalia:** cercus with 14 or more segments, tapered. Sternum VII (subgenital plate) bivalvate apically, valvulae concealed (Figure 7E).

Measurements. Holotype (Figure 6). Body length excluding head 7.3. Maxillary palpomere III–V lengths 1.2/1.5/0.29–0.33. Pronotum length 1.8, width of anterior half 0.82, of posterior half 1.4. Tegmen length 9.0, width 2.4; hindwing length 7.9. Leg segment lengths (femur/tibia//tarsomere 1/2/3/4/5): midleg 3.5/4.2//?/0.50/0.23/0.06/0.21; hindleg 4.0–4.2/6.0–6.4//2.1–2.2/0.65–0.70/0.32/0.07–0.08/0.26–0.27. Stylus length 0.20, interstylar distance ca. 0.56. **Allotype** (Figure 7). Body length excluding head ca. 6.7. Maxillary palpomere II–V lengths 0.72/1.60/1.66/0.44. Lengths of femur/tibia: foreleg 3.0/3.1, midleg 3.4?/3.8, hindleg 4.8/?. Foretarsi four-segmented (regenerated form), tarsomeres 1/2(+3?)/4/5 lengths 1.9/0.80/0.12/0.28 (Figure 7C). Cercus length 1.7 or longer. **Paratype**, NIGP174204 (Figure 8 and Figure 9). Head length ca. 1.4. Maxillary palpomere II–V lengths 0.48/1.08/1.13/0.36. Pronotum length 1.6, width of anterior half 0.82, of posterior half 1.4. Tegmen length 8.2, width 2.6. Hindwing length 7.7, width of prevannus 2.5. Leg segment lengths (femur/tibia//tarsomere 1/2/3/4/5): foreleg 2.6?/2.4//1.3/0.50/0.26/0.07/0.20, midleg 3.0?/3.3//1.3/0.50/0.23/?/?, hindleg 3.7/5.5//1.9/0.60/0.25/0.08/0.27. **Paratype**, NIGP174205 (Figure 10A). Head length ca. 1.7. Maxillary palpomere III–V lengths 1.3/1.5–1.8/0.37–0.47. Pronotum length 2.2, width of anterior half 1.1, of posterior half 1.5.

### 3.3. Undetermined Adults

**Figure 11:**
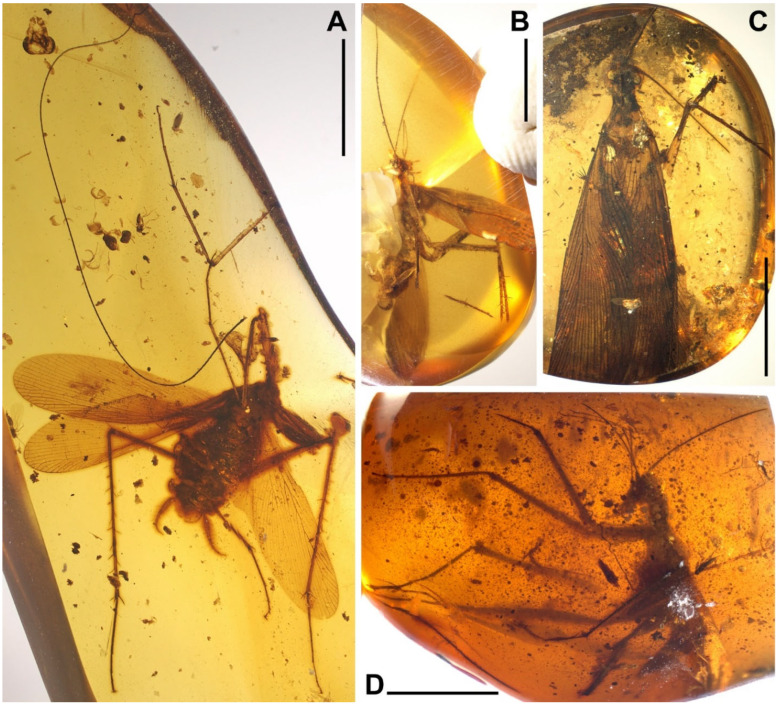
Undetermined adults of Manipulatoridae. (**A**) Female, NIGP174207, dorsal view; note the head at top-left, which may belong to this insect. (**B**) Female, NIGP174208, ventral view. (**C**) Sex unknown, NIGP174209, dorsal view. (**D**) Sex unknown, NIGP174210, lateral view. Scale bars = 5 mm.

NIGP174207 (Figure 11A). This specimen essentially conforms to the description of *Manipulator modificaputis*, but the row of spines of forefemora are too short compared with determined specimens of *Manipulator modificaputis*. The forefemoral spination exhibits an intermediate type between *Manipulator* and *Manipulatoides*.

NIGP174208 (Figure 11B). Too poorly preserved to identify.

NIGP174209 (Figure 11C). Too poorly preserved to identify.

NIGP174210 (Figure 11D). Too poorly preserved to identify.

### 3.4. Undetermined Nymphs

**Figure 12:**
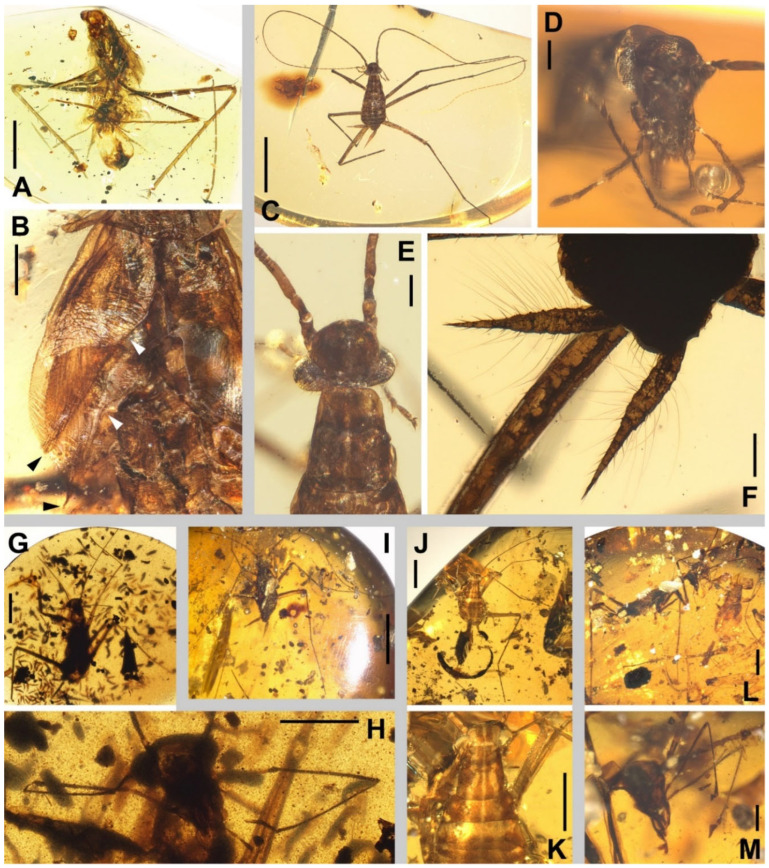
Undetermined nymphs of Manipulatoridae. (**A**,**B**) NIGP174198. (**A**) The specimen in ventral view. (**B**) Close-up of left wing-pads, with black arrowheads indicating the apices and white arrowheads indicating the notch between wing-pads and nota (which are incompletely preserved). (**C**–**F**) NIGP174201. (**C**) The specimen in dorsal view. (**D**) Head in cephalic view. (**E**) Head and pronotum in dorsal view; note the strongly convex frons. (**F**) Cerci, showing long hairs. (**G**,**H**) NIGP174211 in ventral view (**G**) and a close-up of the head (**H**). (**I**) NIGP174212 in dorsal view. (**J**,**K**) NIGP174213 in dorsal view (**J**) and a close-up of the vertex and thorax (**K**). (**L**,**M**) NIGP174363 in lateral view (**L**) and a close-up of the head (**M**). Scale bars: (**A**,**C**,**G**,**I**,**J**,**L**) = 2 mm, (**B**,**M**) = 500 µm, (**D**–**F**) = 200 µm, (**H**,**K**) = 1 mm.

NIGP174198 (Figure 12A,B). Middle-older instar, body length excluding head ca. 5.6. Appendages elongate like adults of this family. Right forefemur incomplete, but several short spines interlaced with spinules are present, which suggests a *Manipulator* species. Wing-pads very large and long (Figure 12B). Spination of mid- and hindlegs similar to adults of *Manipulator modificaputis* but left hindtibia with only 15 lateral spines.

NIGP174201 (Figure 12C–F). Very young instar, body length excluding head 2.0. Antennae and legs elongate like adults of this family, but elongation of maxillary palpi not as significant as in adults (Figure 12D). Head with frons very convex (Figure 12E). Leg spination too weak to exhibit characteristics. Cerci with very long hairs (Figure 12F).

NIGP174211 (Figure 12G,H). Turbid specimen. Body length excluding head ca. 5.4. Vertex with a pair of dark longitudinal stripes. Pronotum with four longitudinal stripes; middle of the inner pair narrow.

NIGP174212 (Figure 12I). Young instar, body length excluding head 2.4. Very similar to NIGP174201 (Figure 12C–F).

NIGP174213 (Figure 12J,K). Body length excluding head ca. 3.3. Vertex with a pair of dark longitudinal stripes (Figure 12K). Three nota with four longitudinal stripes, of which the inner pair are extended from vertex, and the outer pair fill the lateral borders of the nota (Figure 12K). Wing-pads initially developed. Leg spination similar to the adults of *Manipulatoides obscura*, but it is unclear whether it is due to species identity or young instar.

NIGP174363 (Figure 12L,M). Older instar, male, body length excluding head ca. 6.4. Wing-pads obviously developed. Leg spination similar to the adults of *Manipulatoides obscura*.

## 4. Discussion

### 4.1. Comment on Original Description and Character States of Manipulator modificaputis

Vršanský and Bechly (hereafter VB15) described the holotype in detail and listed the characters in phylogenetic context [4]. However, the following descriptions are open to debate, as are the corresponding phylogenetic statements.

Head. (1) The eyes were considered to be “extremely large”. This is somewhat exaggerated. The ocular size in relation to head can be largely determined by the interocular distance, and that of *Manipulator* is of moderate size among cockroaches. VB15 put this character into family-level phylogeny. However, relative size of eyes is considerably variable in cockroaches, and large eyes are present in phylogenetically distant genera, e.g., *Eupolyphaga* and *Magniocula* of two corydiid subfamilies [21,22], *Panchlora* and *Pseudoglomeris* of two blaberid subfamilies [23,24], of which the relative sizes of eyes are all larger than the cockroaches mentioned in VB15. (2) The modifications of antennomeres suggested by VB15 cannot be justified based on our materials or the figures in VB15. Original description of antennae includes “segments 1–3 extremely modified”, “segments 4–13 short”, and “segment 11 extremely short and modified”. However, the antennae of *Manipulator* are normal in cockroaches, except for elongation to some extent. A straightforward explanation for the morphology mentioned in VB15 is that the third segment (first flagellomere) is the meriston and the fourth through thirteenth segments are meristal segments [25]. (3) VB15 described an “invisible central ocellus” whose position is “visible” but stated “central ocellus missing” in the character list. In our materials of *Manipulator modificaputis*, the frons is barely observable. Nevertheless, it is clear that *Manipulatoides obscura* does not has a central ocellus. Furthermore, the central ocellus is absent from all extant cockroaches, and Manipulatoridae belong to extant superfamily Corydioidea. Therefore, it is implausible for *Manipulator modificaputis* to have a central ocellus. (4) According to the measurements in VB15, the first maxillary palpomere is elongate, but this is not seen in their figures or our materials (see normal length of the first palpomere in Figure 1A). (5) VB15 described “terminal palpomere extremely small, but elongated with ventral cavity”, and considered it as an autapomorphy. However, the terminal palpomere is of normal size in relation to the head; what is observed is the elongation of other palpomeres rather than the reduction of the terminal palpomere. The cavity in this palpomere is the sensory field, a common structure in cockroaches (see Section 4.2 below for details). Therefore, the terminal palpomere of *Manipulator modificaputis* is ordinary and plesiomorphic.

Thorax. (1) The oblique vein basally between RP and M in the hindwing, considered herein to be an autapomorphy and also a diagnostic character of this family, was not mentioned in VB15. This special vein is noticeable in their Figure 2A, but omitted from the drawing in their Figure 1D, in which the first branch of RP seems omitted or mis-illustrated. (2) Almost as a rule, the hindleg of cockroaches is longer than the midleg, and the latter longer than the foreleg, with roughly the same segmentation ratio. Our materials are no exceptions, but the measurements in VB15 appear anomalous and merit a clarification. (3) VB15 described the walking legs without spines, but this is neither supported by their figures nor by our materials. (4) “Terminal claw symmetrical, large”—this was regarded as an autapomorphy by VB15. However, the claws of *Manipulator* are normal in cockroaches, and such claws are symplesiomorphic for most cockroaches.

Abdomen. VB15 described segmented styli without photo or illustration. In our materials, all styli are not segmented; this is consistent with the taxonomic placement of Manipulatoridae in extant superfamily Corydioidea. At least in Blattaria, a stylus is always unsegmented, as its definition (see [14], “style”).

### 4.2. Ethology

Manipulatoridae are impressive by the elongate pronotum and appendages, especially for the enormously elongate maxillary palpi, which surely suggest particular adaptation. Vršanský and Bechly suggested that *Manipulator* is a predator, based on the “freely movable head”, “semi-raptorial forelegs”, and elongate appendages [4]. Extant cockroaches are detritivores in general, but some of them are also considered as generalist predators, i.e., they feed on weak, soft-bodied small animals occasionally [26,27]. Accordingly, it is not surprising that Mesozoic cockroaches also feed on alive small animals sometimes. However, it is implausible that *Manipulator* cockroaches are obligate predators, because they lack necessary weaponry for specialized predation. Compared with the type-A spination of extant cockroaches (e.g., [28,29,30,31]), the forefemoral spination of *Manipulator* is weak. It is unjustified to interpret the forelegs of type A as raptorial, not to mention that of *Manipulator*. The forefemoral spination of *Manipulatoides* is even weaker than that of *Manipulator*, lacking spines along the ventral margin of the forefemur (type C), and is not competent to capture prey at all. In comparison, mantises, the predatory relatives of cockroaches, possess forelegs particularly specialized for predation, which are obviously stronger and able to engage with the specialized tibia [32,33]. In addition, *Manipulator* and *Manipulatoides* do not possess very strong mandibles or other morphological specialization for capturing. In view of the similar body plan and close relationship between these two genera, their feeding habit is likely the same; both genera are unlikely genuine hunters.

Apart from Manipulatoridae, some fossil cockroaches were also reported as predators. A comparison between those cockroaches and Manipulatoridae is supposed to be beneficial, but in many cases, the predatism of the former remains highly conjectural, while in other cases, the evidence appears not strong. Among them, *Raptoblatta waddingtonae* Dittmann et al., 2015 might be the most plausible [34]. *Raptoblatta waddingtonae* is a Cretaceous insect in the typical habitus of cockroaches. It possesses opposing spines on the foreleg femur and tibia, which is reminiscent of mantises, but on the other hand, such a foreleg is also found in detritivorous extant cockroaches (e.g., *Periplaneta banksi* Hanitsch, 1931 in [35]). *Stavba babkaeva* Vršanská and Vršanský, 2019 is another fossil that was interpreted as a predatory cockroach; its predatism was judged mainly from the mantis-like head with large eyes and the foreleg spination [36]. The head is indeed somewhat similar to that of mantises, but it indicates an excellent visual system rather than predatism (even if related, indirectly), and such heads are found, at least in extant Pseudophyllodromiinae (e.g., [37,38,39]), of which no species was reported as an obligate predator. In addition, large eyes are also found among extant Corydiidae that inhabit soil and sand (e.g., [21,40]). The forefemoral spination of *Stavba babkaeva* is of the normal type-B of cockroaches (see [14]), and is irrelevant to predation. Type-B spination is common within cockroaches, and no species of this type was reported as an obligate predator. Raphidiomimidae, the most comparable fossil family, resemble Manipulatoridae in habitus to a great extent (see Section 3), and this family was also considered to be predators based on elongate habitus, modified prognathous head and spinose forelegs [41,42,43] (but note the species described in [43] are far different from Raphidiomimidae in the ovipositor). This reasoning is similar to that for Manipulatoridae [4]. As stated above, these traits are not concrete evidence for the adaptation of predation, and such habitus warrants alternative interpretations.

The feeding habit of Manipulatoridae other than predation was briefly discussed by the authors of the present paper [44], here we elaborate on this issue. First, extraordinarily elongate palpi are rare, two examples comparable with that of Manipulatoridae are found in Coleoptera and one in Polyneoptera. *Clidicostigus* Jałoszyński et al., 2017, an ant-like stone beetle, is from Myanmar amber too [45]. Jałoszyński et al. speculate that the elongate maxillary palpi are adapted for detecting prey or danger with longer organs of tactile and chemical senses [45]. *Clidicostigus* has sharp, powerful mandibles, which are an adaptation for predation. In comparison, *Leptopalpus rostratus* (Fabricius, 1792), an extant blister beetle, uses the long maxillary palps for nectar uptake [46]. In addition to elongation, the maxillary palpi of *L. rostratus* are also modified with a row of bristles. In Manipulatoridae, other modifications that can synergize the elongate maxillary palpi are not detected; therefore, it is implausible that the maxillary palpi alone are adapted for prey detecting or nectar feeding. Elongate palpi, presumedly maxillary, are also found in the enigmatic polyneopteran *Eucaenus* Scudder, 1885 from Carboniferous [47], but the function is unknown.

The maxillary palpi of cockroaches possess a sensory field on the terminal palpomere, which is densely filled with sensilla [48,49,50]. These sensilla are likely used for smelling [49]; this inference conforms to the fact that the cockroaches use the palpi to verify potential food. Longer palpi allow the cockroaches to inspect the food at a greater distance and might suggest a certain food source that is rare in the menu of extant cockroaches.

In addition to the maxillary palpi, the antennae and legs of Manipulatoridae are also elongate, although not that remarkable. Cockroaches with elongate appendages are found mainly in caves [27], alike many troglobitic arthropods [51]. Normally, cave dwellers have a set of morphological modifications including eye loss, pigmentation loss, and wing loss [51,52], e.g., the cavernicolous cockroach *Nocticola* Bolívar, 1892 [53,54]. None of these modifications is found in Manipulatoridae; in contrast, the eyes and wings of Manipulatoridae are well developed. Furthermore, the occurrence in amber almost rules out the possibility of being a cave dweller.

**Figure 13:**
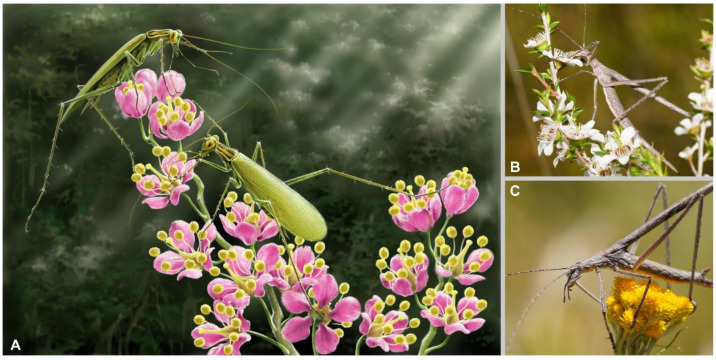
(**A**) Ethological reconstruction of *Manipulator modificaputis*, drawn by Mr Jie Sun. (**B**,**C**) *Zaprochilus australis*, shared by Reiner Richter © CC BY-NC-SA 3.0 ((**B**) available from https://images.ala.org.au/image/details?imageId=78af80ab-fd6b-4de0-8ee0-7374845fd129, accessed on 14 July 2018; (**C**) available from https://www.inaturalist.org/observations/66843712, accessed on 23 January 2021).

The long legs may function as brackets for stretching the body to stride across discrete spaces, and the long antennae and palpi as probes for detecting foods at a distance. Based on these modifications together, we speculate that Manipulatoridae feed on flower tissue (Figure 13A). Plant litter is the primary food source of most extant cockroaches [26,27], it is so readily accessible that these detritivorous cockroaches do not require a powerful sense organ to forage. Flower as a food source is far different from the plant litter: flowers are food islands amid branches and twigs, they are far separated, and detecting flowers requires powerful sense organs. The modifications of Manipulatoridae can be useful in such cases: the long antennae are helpful for detecting a distant food source, the well-developed wings support frequent long-distance movement, and the long legs are used to stride over the gaps or barriers on plant surfaces. With the long legs, Manipulatoridae can efficiently travel from one branch to another without walking through the entire branches, or from one leaf to another without climbing over both sides of a leaf. Some protective structure of plants, e.g., acanthae, can be readily overcame. Then Manipulatoridae stretch the long maxillary palpi into the corolla to verify the food, probably via nectar. Finally, these cockroaches may enjoy the feast when the plant is recognized. Occasional herbivory was reported from a variety of extant cockroaches [26,27], which are essentially in the typical morphotype of most cockroaches. This implies that occasional herbivory may not require adaptive modifications to morphotype. In other words, the morphotype of Manipulatoridae may not be adequately accounted for by occasional herbivory. The advantage of long-appendaged morphotype may be great energy save when foraging for flowers. Comparable morphotype is found in extant Zaprochilinae of Orthoptera, which are obligate pollen- and nectar-feeders (Figure 13B,C) [55,56]. Zaprochilinae share many morphological adaptations with Manipulatoridae: long and agile body, nimble head, narrow saddle-like pronotum, and elongate legs. The maxillary palpi of Zaprochilinae are not that long as that of Manipulatoridae, but still remarkable. Manipulatoridae occurred during the radiation of angiosperm, which provided new niches and allowed the co-radiation of flower-related organisms. Judging from the shape and size, some flowers from the Myanmar amber might be the host plants of Manipulatoridae (e.g., [57,58]).

In an alternative scenario, the Manipulatoridae are carnivorous kleptoparasites. An animal that has to keep a certain distance from its potential food source can be a spider-web dweller. Emesinae, or thread-legged bugs, of the hemipteran family Reduviidae, are of that kind. They have elongate bodies (especially pronota) and legs, frequently found on spider webs, feeding on the web owner or the small animals (mainly insects) entangled in the web [59]. Manipulatoridae seem to be manoeuvrable enough to wander about a spider web, but it would seem almost impossible for them to capture a living spider since they lack specialized raptorial structures. Furthermore, they seem to be unable to walk on silks, since their legs, especially tarsi, are typical (except for the length) of cockroaches (cp. Emesinae in [59] and *Pedanoptera* in [60]). If Manipulatoridae are spider-web kleptoparasites, they might stand outside the spider web when foraging, smell and palpate with the long antennae and maxillary palpi, reach out, and steal the spider’s prey or even eggs, and quickly escape from the web owner. In addition, spider silks were found in Early Cretaceous ambers [61,62], and web-spinning spiders had originated by the Cretaceous period [63]. These facts allow web-dwelling cockroaches to exist in the Cretaceous period.

### 4.3. Regeneration of Tarsus

Four-segmented foretarsi are found from the allotype of *Manipulatoides obscura* gen. & sp. nov. (Figure 7C). A normal tarsus of a cockroach has five segments (tarsomeres), whilst the four-segmented tarsus is due to the regrowth of an impaired predecessor [64,65,66,67]. In the regenerated tarsus, the third tarsomere is either disappeared or has only a little component left [66,67]; therefore, we label the tarsomere as “t2(+3?)” in Figure 7C. Regeneration was documented across extant cockroach families [68], and the regeneration capabilities are well-developed [69]. This suggests that the regeneration is plesiomorphic for Blattodea. Four-segmented regenerated tarsi have been reported from a fossil cockroach, namely, *Piniblattella magna* Lee, 2016 (Ectobiidae) from the Aptian Crato Formation of Brazil [70]. *Manipulatoides obscura* gen. & sp. nov. is thus another example. Four-segmented tarsi of non-Blattodean cockroaches were also mentioned in the description of *Antophiloblatta hispida* Sendi, 2020 and *Alienopterix mlynskyi* Sendi, 2021 [71,72]. In addition, Ping described *Cainoblattinopsis fushunensis* from the Fushun amber of north-eastern China and established a new family for it [73]. However, this species and the family Cainoblattinidae are characterized by the four-segmented tarsus of the right foreleg. *Cainoblattinopsis fushunensis* has to be reinvestigated and the family is unlikely to be valid.

## 5. Conclusions

This paper revisits the taxonomy and ethology of the fossil cockroach family Manipulatoridae from Cretaceous. The known species *Manipulator modificaputis* is redescribed and a new genus and species *Manipulatoides obscura* is proposed. Manipulatoridae belong in the extant superfamily Corydioidea, but are distinct from other members of this superfamily in the elongation of pronotum and appendages, which suggests particular adaptation. In terms of feeding habit, the evidence provided herein is in favour of the flower-feeding hypothesis over the previous predation hypothesis. Nevertheless, the habit of Manipulatoridae can be variously interpreted and is open to debate, e.g., the kleptoparasite hypothesis is also regarded as a less probable scenario in this paper.

## Data Availability

All data are provided in the manuscript.
